# Supplementary material for: Viral and host small RNA transcriptome analysis of SARS-CoV-1 and SARS-CoV-2-infected human cells reveals novel viral short RNAs
Source: Heliyon. 2024 Jan 17;10(3):e24570. doi: 10.1016/j.heliyon.2024.e24570 (PMC10837498; doi:10.1016/j.heliyon.2024.e24570)
Supplement: Multimedia component 1 [file mmc1.pptx]

## Slide 1
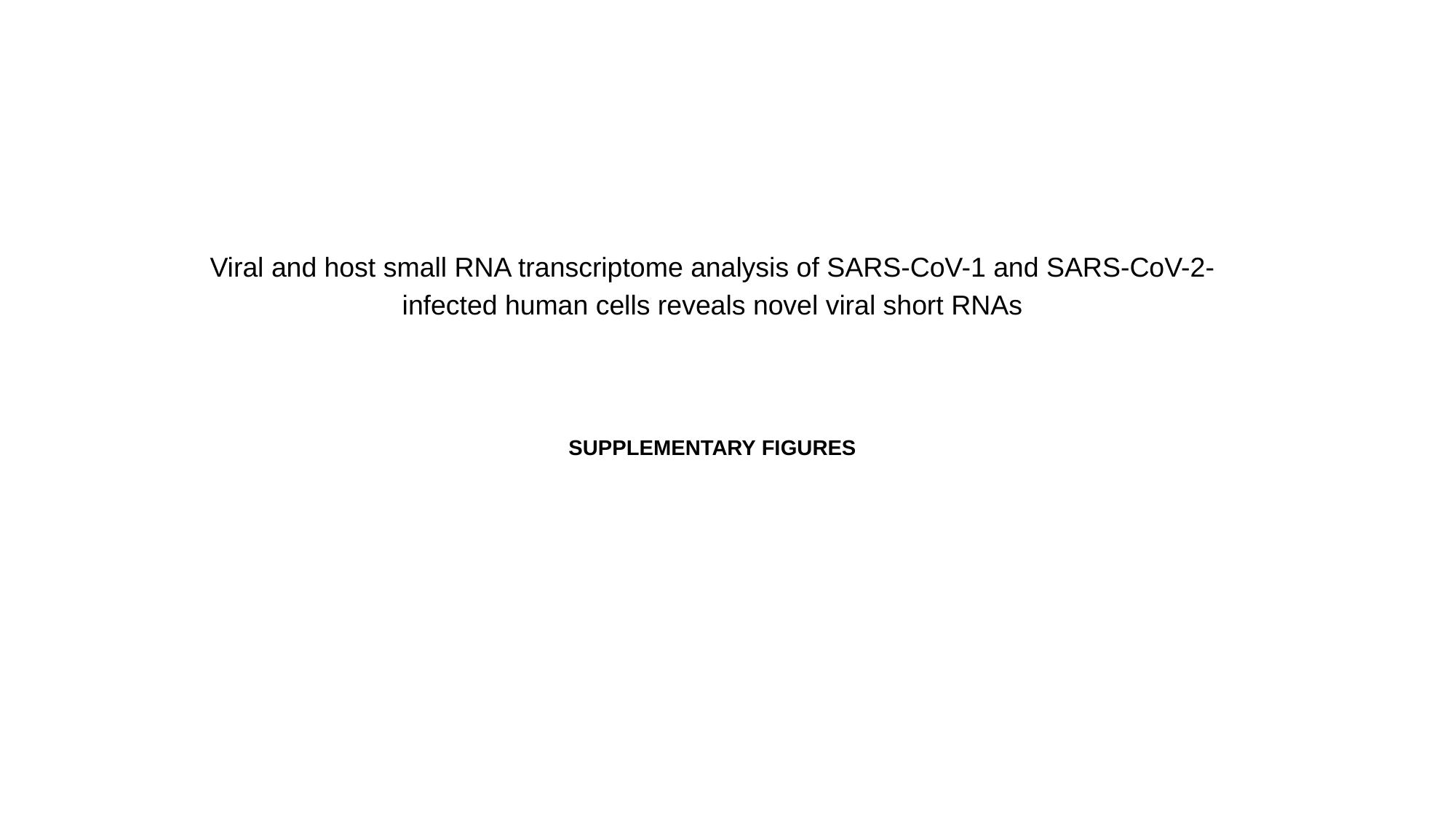

Viral and host small RNA transcriptome analysis of SARS-CoV-1 and SARS-CoV-2-infected human cells reveals novel viral short RNAs
SUPPLEMENTARY FIGURES

## Slide 2
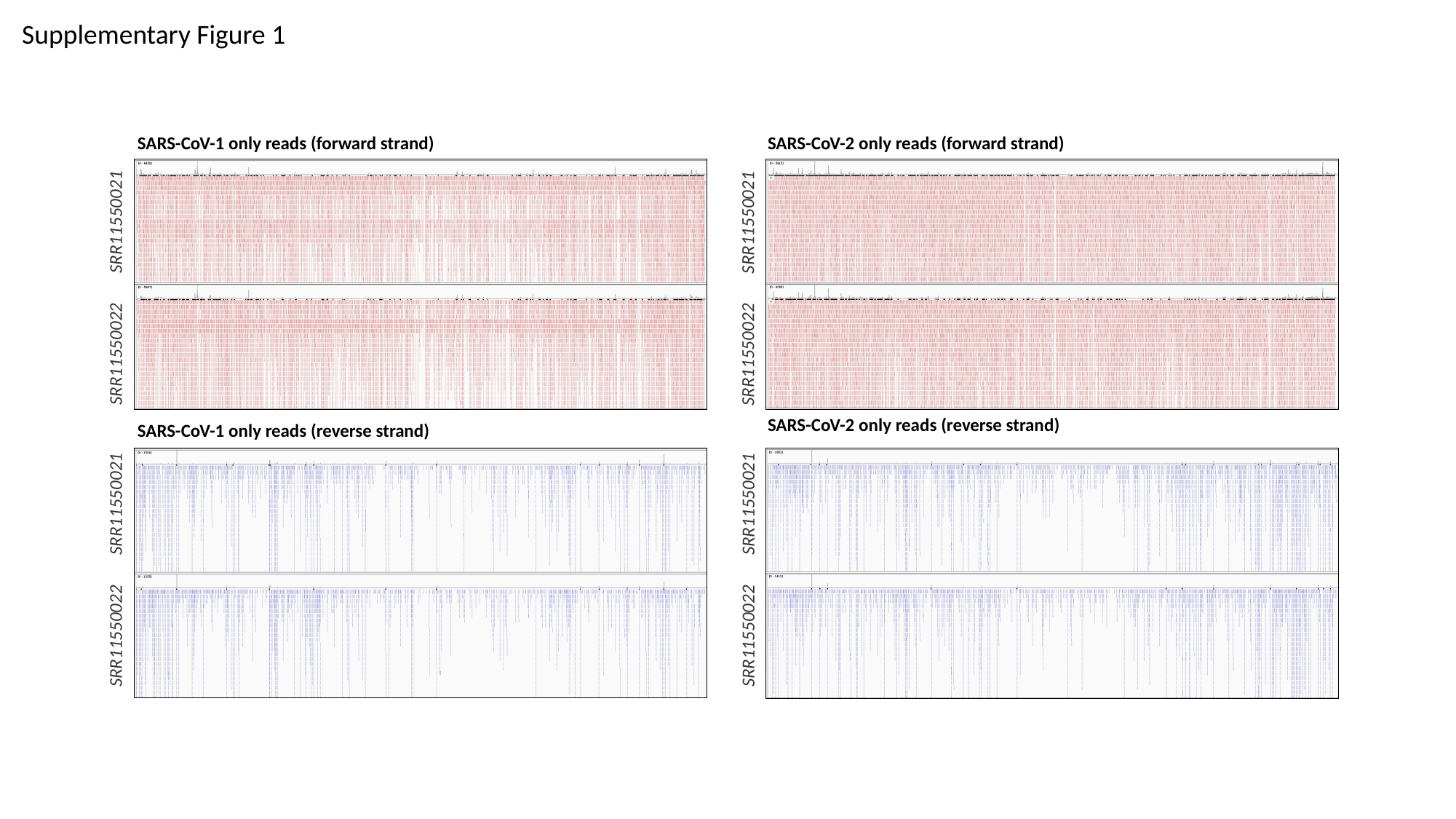

Supplementary Figure 1
SARS-CoV-1 only reads (forward strand)
SARS-CoV-2 only reads (forward strand)
SRR11550021
SRR11550021
SRR11550022
SRR11550022
SARS-CoV-2 only reads (reverse strand)
SARS-CoV-1 only reads (reverse strand)
SRR11550021
SRR11550021
SRR11550022
SRR11550022

## Slide 3
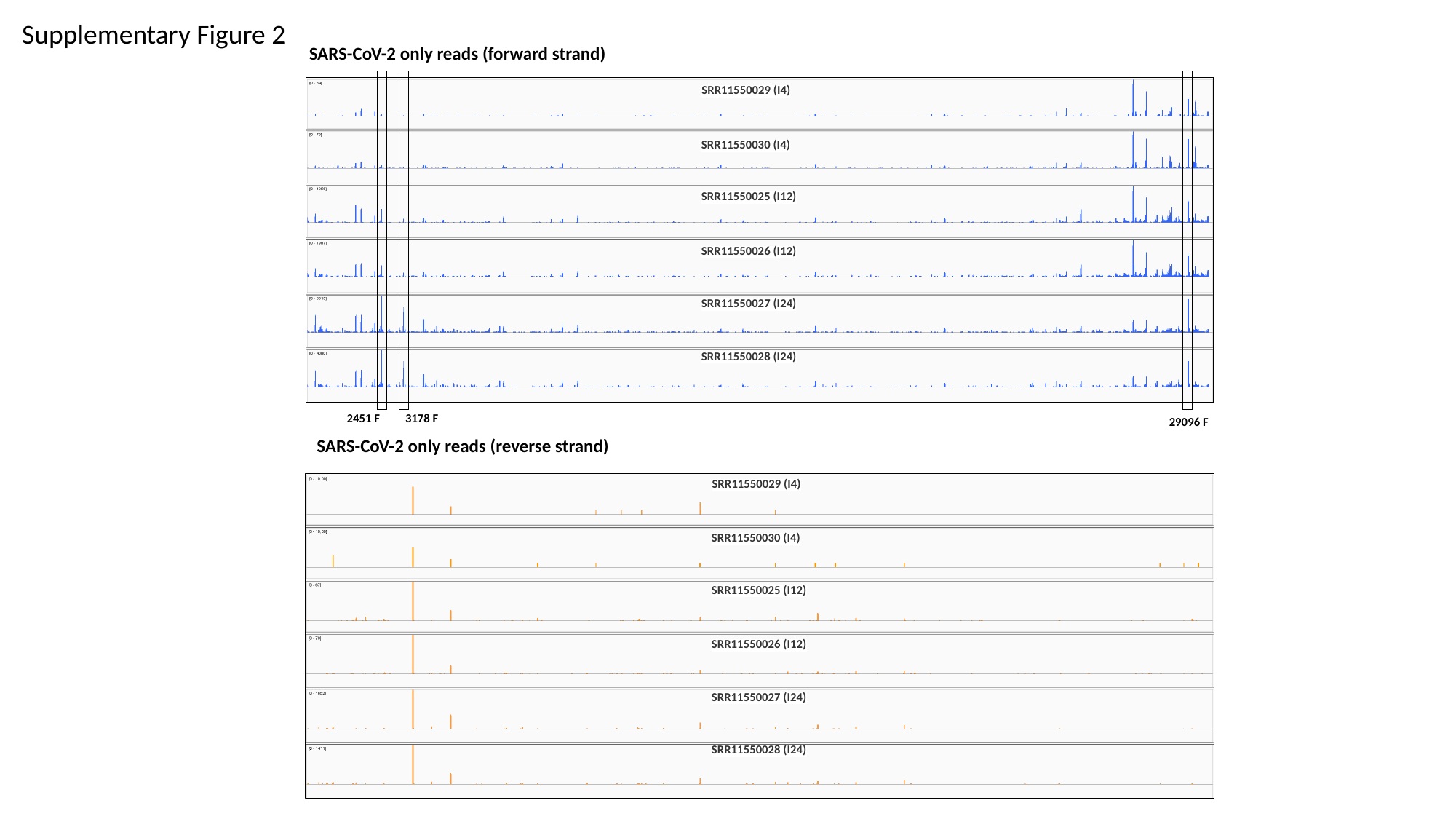

Supplementary Figure 2
SARS-CoV-2 only reads (forward strand)
SRR11550029 (I4)
SRR11550030 (I4)
SRR11550025 (I12)
SRR11550026 (I12)
SRR11550027 (I24)
SRR11550028 (I24)
3178 F
2451 F
29096 F
SARS-CoV-2 only reads (reverse strand)
SRR11550029 (I4)
SRR11550030 (I4)
SRR11550025 (I12)
SRR11550026 (I12)
SRR11550027 (I24)
SRR11550028 (I24)

## Slide 4
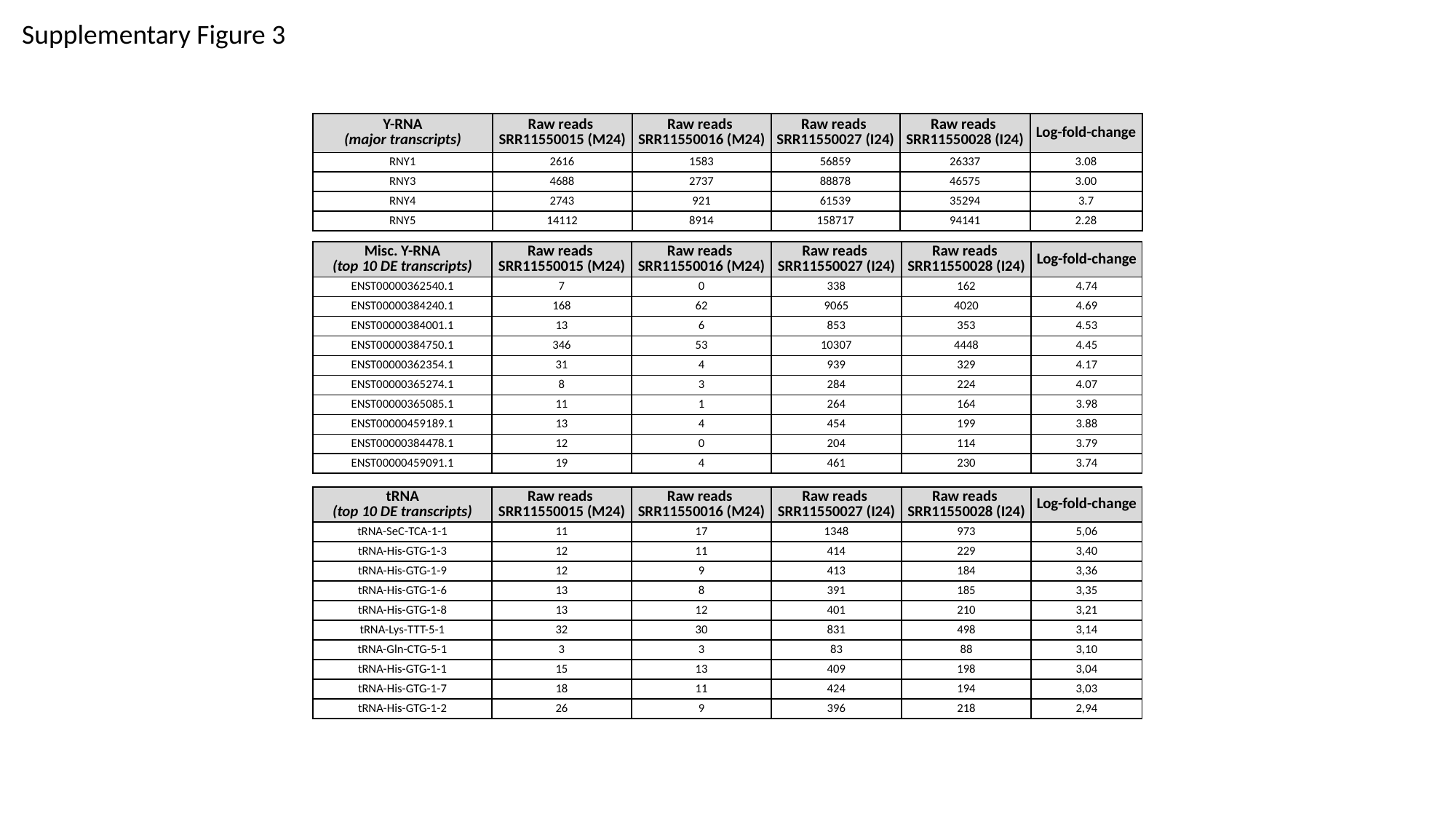

Supplementary Figure 3
| Y-RNA (major transcripts) | Raw reads SRR11550015 (M24) | Raw reads SRR11550016 (M24) | Raw reads SRR11550027 (I24) | Raw reads SRR11550028 (I24) | Log-fold-change |
| --- | --- | --- | --- | --- | --- |
| RNY1 | 2616 | 1583 | 56859 | 26337 | 3.08 |
| RNY3 | 4688 | 2737 | 88878 | 46575 | 3.00 |
| RNY4 | 2743 | 921 | 61539 | 35294 | 3.7 |
| RNY5 | 14112 | 8914 | 158717 | 94141 | 2.28 |
| Misc. Y-RNA (top 10 DE transcripts) | Raw reads SRR11550015 (M24) | Raw reads SRR11550016 (M24) | Raw reads SRR11550027 (I24) | Raw reads SRR11550028 (I24) | Log-fold-change |
| --- | --- | --- | --- | --- | --- |
| ENST00000362540.1 | 7 | 0 | 338 | 162 | 4.74 |
| ENST00000384240.1 | 168 | 62 | 9065 | 4020 | 4.69 |
| ENST00000384001.1 | 13 | 6 | 853 | 353 | 4.53 |
| ENST00000384750.1 | 346 | 53 | 10307 | 4448 | 4.45 |
| ENST00000362354.1 | 31 | 4 | 939 | 329 | 4.17 |
| ENST00000365274.1 | 8 | 3 | 284 | 224 | 4.07 |
| ENST00000365085.1 | 11 | 1 | 264 | 164 | 3.98 |
| ENST00000459189.1 | 13 | 4 | 454 | 199 | 3.88 |
| ENST00000384478.1 | 12 | 0 | 204 | 114 | 3.79 |
| ENST00000459091.1 | 19 | 4 | 461 | 230 | 3.74 |
| tRNA (top 10 DE transcripts) | Raw reads SRR11550015 (M24) | Raw reads SRR11550016 (M24) | Raw reads SRR11550027 (I24) | Raw reads SRR11550028 (I24) | Log-fold-change |
| --- | --- | --- | --- | --- | --- |
| tRNA-SeC-TCA-1-1 | 11 | 17 | 1348 | 973 | 5,06 |
| tRNA-His-GTG-1-3 | 12 | 11 | 414 | 229 | 3,40 |
| tRNA-His-GTG-1-9 | 12 | 9 | 413 | 184 | 3,36 |
| tRNA-His-GTG-1-6 | 13 | 8 | 391 | 185 | 3,35 |
| tRNA-His-GTG-1-8 | 13 | 12 | 401 | 210 | 3,21 |
| tRNA-Lys-TTT-5-1 | 32 | 30 | 831 | 498 | 3,14 |
| tRNA-Gln-CTG-5-1 | 3 | 3 | 83 | 88 | 3,10 |
| tRNA-His-GTG-1-1 | 15 | 13 | 409 | 198 | 3,04 |
| tRNA-His-GTG-1-7 | 18 | 11 | 424 | 194 | 3,03 |
| tRNA-His-GTG-1-2 | 26 | 9 | 396 | 218 | 2,94 |

## Slide 5
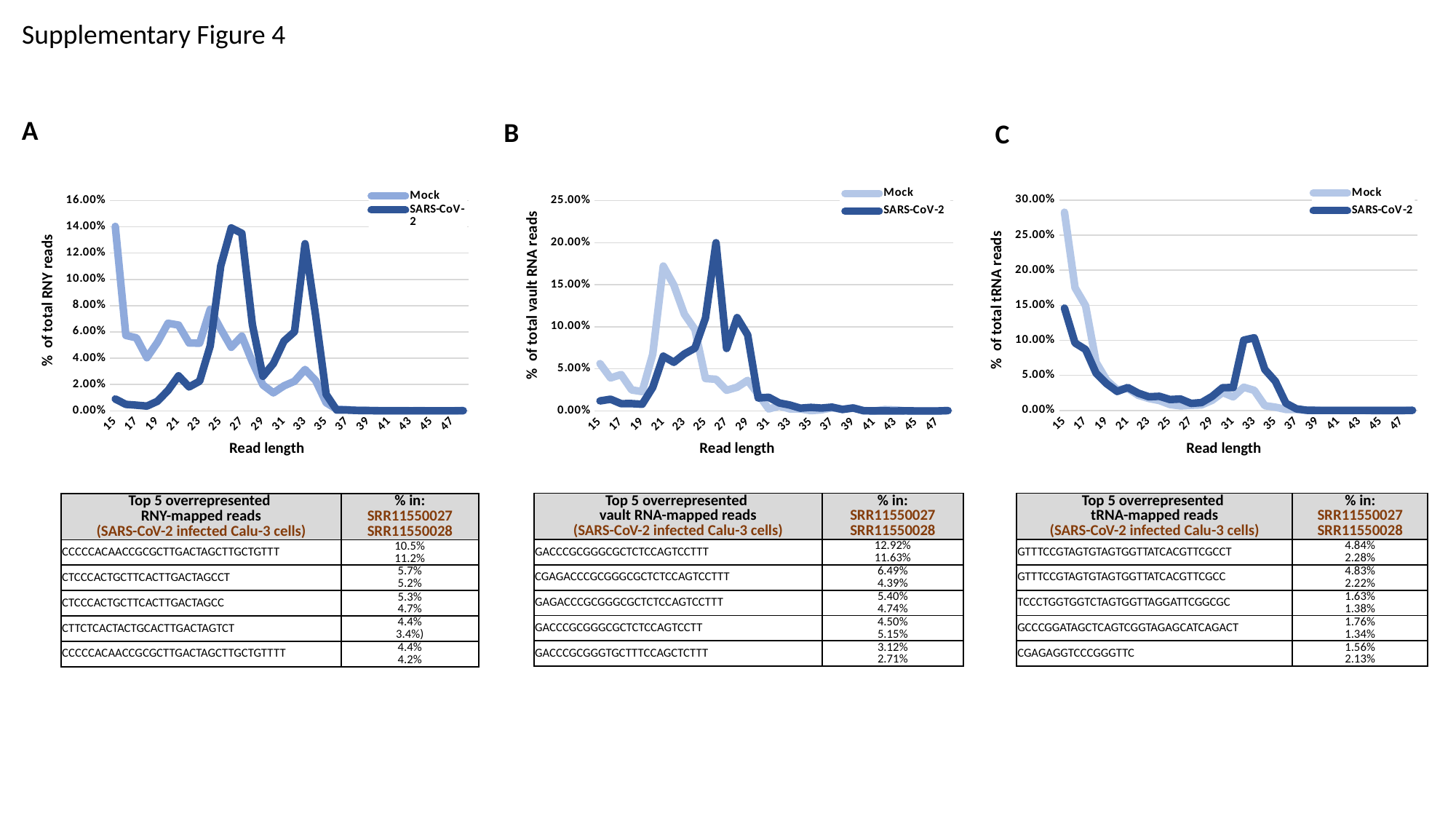

Supplementary Figure 4
A
B
C
### Chart
| Category | Mock | SARS-CoV-2 |
|---|---|---|
| 15 | 0.28274914653265215 | 0.14597153421330733 |
| 16 | 0.17512878218602365 | 0.09622016814743765 |
| 17 | 0.1494296356646351 | 0.08689926939511772 |
| 18 | 0.06851102015733015 | 0.053005052307192185 |
| 19 | 0.04319283364102373 | 0.0380988414092884 |
| 20 | 0.029533106632205014 | 0.02714262361711418 |
| 21 | 0.031422818575058756 | 0.03270384940388345 |
| 22 | 0.021688368623267916 | 0.024699685760211115 |
| 23 | 0.017048616843166 | 0.019548602131161347 |
| 24 | 0.014215805024004848 | 0.020178697378272586 |
| 25 | 0.008488375029175241 | 0.015596412028835717 |
| 26 | 0.006581696033184894 | 0.016310275622775608 |
| 27 | 0.007392576794449159 | 0.010065393402172836 |
| 28 | 0.008006160525033735 | 0.01124099843820664 |
| 29 | 0.014505386645145439 | 0.020086518648176455 |
| 30 | 0.025586858829960618 | 0.03249253398578911 |
| 31 | 0.019429206010109088 | 0.03263005232089845 |
| 32 | 0.033145041025965855 | 0.10027337797591204 |
| 33 | 0.02864744591375507 | 0.10359698378821362 |
| 34 | 0.006845586443510905 | 0.05840572143022667 |
| 35 | 0.004665806497486409 | 0.04161632863116444 |
| 36 | 0.0015025108883529197 | 0.010312400665645455 |
| 37 | 0.0013541746087732383 | 0.002271909060574952 |
| 38 | 0.00017299840841464257 | 0.0002771104011819117 |
| 39 | 0.00012846117388374658 | 9.336200755549417e-05 |
| 40 | 6.919936336585703e-05 | 7.042043972874889e-05 |
| 41 | 3.4599681682928514e-05 | 3.165258126800241e-05 |
| 42 | 0.0 | 5.73539195668632e-06 |
| 43 | 6.919936336585703e-05 | 7.913145317000602e-06 |
| 44 | 0.0 | 5.73539195668632e-06 |
| 45 | 3.4599681682928514e-05 | 0.0 |
| 46 | 0.0 | 0.0 |
| 47 | 0.0 | 0.0 |
| 48 | 0.00041998320333416826 | 0.00014084087945749778 |
### Chart
| Category | Mock | SARS-CoV-2 |
|---|---|---|
| 15 | 0.14033555131610786 | 0.008937309478790002 |
| 16 | 0.05731540933871678 | 0.00484824527064516 |
| 17 | 0.05549353366280898 | 0.004256122557463277 |
| 18 | 0.04036925596347604 | 0.0035329173032405017 |
| 19 | 0.05207015502748781 | 0.007248129038522095 |
| 20 | 0.0665692328650022 | 0.01546752062152777 |
| 21 | 0.06521280795899893 | 0.026582369555008672 |
| 22 | 0.05151875389772125 | 0.018132802772952556 |
| 23 | 0.051421397588088294 | 0.02251019230031171 |
| 24 | 0.07716695683046129 | 0.049340100292587216 |
| 25 | 0.062231557916323496 | 0.11024139740111917 |
| 26 | 0.048287877165597064 | 0.13912696027096355 |
| 27 | 0.05677896855737427 | 0.1350300289146325 |
| 28 | 0.03707652596906381 | 0.06555817718828234 |
| 29 | 0.019591675770883783 | 0.026244600387851334 |
| 30 | 0.013624486686569253 | 0.03591174110652401 |
| 31 | 0.018956950237643936 | 0.05297856309720659 |
| 32 | 0.022462077117759625 | 0.060118469022077264 |
| 33 | 0.03140533305132082 | 0.12713646097702322 |
| 34 | 0.023059779005300297 | 0.0727457025902506 |
| 35 | 0.006122500660838886 | 0.012464483062508455 |
| 36 | 0.001188242341678187 | 0.0005454427617178197 |
| 37 | 0.0008278488348027651 | 0.0005441960089471467 |
| 38 | 0.0002483546504408295 | 0.0002536550287882461 |
| 39 | 0.00015950053256764187 | 0.00017482172690585026 |
| 40 | 4.139244174013825e-05 | 2.3284184688235926e-05 |
| 41 | 8.27848834802765e-05 | 1.398245017058038e-05 |
| 42 | 9.741186995743451e-05 | 1.3138883647065962e-05 |
| 43 | 2.0696220870069125e-05 | 1.3661463470612826e-06 |
| 44 | 4.139244174013825e-05 | 8.779154694108682e-06 |
| 45 | 0.0 | 1.3661463470612826e-06 |
| 46 | 0.0 | 0.0 |
| 47 | 0.0 | 0.0 |
| 48 | 0.00022158919517784926 | 7.67429825882083e-06 |
### Chart
| Category | Mock | SARS-CoV-2 |
|---|---|---|
| 15 | 0.05627218330872884 | 0.011946027915909683 |
| 16 | 0.03920831108458712 | 0.013914026854007066 |
| 17 | 0.043255488896566885 | 0.008656122878679836 |
| 18 | 0.024937180010679398 | 0.008712174990627023 |
| 19 | 0.02298976034174074 | 0.007965349001082585 |
| 20 | 0.06737129754687941 | 0.02796628880899629 |
| 21 | 0.1721648553569746 | 0.06505187719602284 |
| 22 | 0.1496619499324685 | 0.057811013665168526 |
| 23 | 0.11492799258724126 | 0.0676923006638297 |
| 24 | 0.09680678141784715 | 0.07460084269442929 |
| 25 | 0.038479599208468135 | 0.11048820109318938 |
| 26 | 0.037643308100637624 | 0.19991546749118078 |
| 27 | 0.02454298457769262 | 0.0742729139929165 |
| 28 | 0.028157097088293497 | 0.11085483662704546 |
| 29 | 0.036305242328108805 | 0.09031990820406122 |
| 30 | 0.019925322737695133 | 0.015404290102637868 |
| 31 | 0.0024193548387096775 | 0.01621095856190111 |
| 32 | 0.005728790401105632 | 0.00949271257280248 |
| 33 | 0.0024193548387096775 | 0.0070130655389507215 |
| 34 | 0.0024193548387096775 | 0.003282525135343384 |
| 35 | 0.0004032258064516129 | 0.00423480859747525 |
| 36 | 0.00129330653013789 | 0.0033758783840364386 |
| 37 | 0.0036290322580645163 | 0.004511077985782794 |
| 38 | 0.0020161290322580645 | 0.0017701221810531914 |
| 39 | 0.003393064673179006 | 0.0035476242639928463 |
| 40 | 0.0004032258064516129 | 0.000227797991903595 |
| 41 | 0.0 | 6.722237160527024e-05 |
| 42 | 0.0016129032258064516 | 9.335324869305452e-05 |
| 43 | 0.0012096774193548388 | 0.0 |
| 44 | 0.0 | 0.00018670649738610905 |
| 45 | 0.0 | 0.0 |
| 46 | 0.0 | 0.0 |
| 47 | 0.0 | 0.0 |
| 48 | 0.0004032258064516129 | 0.000414504489289704 |% of total vault RNA reads
% of total tRNA reads
% of total RNY reads
Read length
Read length
Read length
| Top 5 overrepresented vault RNA-mapped reads (SARS-CoV-2 infected Calu-3 cells) | % in: SRR11550027 SRR11550028 |
| --- | --- |
| GACCCGCGGGCGCTCTCCAGTCCTTT | 12.92% 11.63% |
| CGAGACCCGCGGGCGCTCTCCAGTCCTTT | 6.49% 4.39% |
| GAGACCCGCGGGCGCTCTCCAGTCCTTT | 5.40% 4.74% |
| GACCCGCGGGCGCTCTCCAGTCCTT | 4.50% 5.15% |
| GACCCGCGGGTGCTTTCCAGCTCTTT | 3.12% 2.71% |
| Top 5 overrepresented tRNA-mapped reads (SARS-CoV-2 infected Calu-3 cells) | % in: SRR11550027 SRR11550028 |
| --- | --- |
| GTTTCCGTAGTGTAGTGGTTATCACGTTCGCCT | 4.84% 2.28% |
| GTTTCCGTAGTGTAGTGGTTATCACGTTCGCC | 4.83% 2.22% |
| TCCCTGGTGGTCTAGTGGTTAGGATTCGGCGC | 1.63% 1.38% |
| GCCCGGATAGCTCAGTCGGTAGAGCATCAGACT | 1.76% 1.34% |
| CGAGAGGTCCCGGGTTC | 1.56% 2.13% |
| Top 5 overrepresented RNY-mapped reads (SARS-CoV-2 infected Calu-3 cells) | % in: SRR11550027 SRR11550028 |
| --- | --- |
| CCCCCACAACCGCGCTTGACTAGCTTGCTGTTT | 10.5% 11.2% |
| CTCCCACTGCTTCACTTGACTAGCCT | 5.7% 5.2% |
| CTCCCACTGCTTCACTTGACTAGCC | 5.3% 4.7% |
| CTTCTCACTACTGCACTTGACTAGTCT | 4.4% 3.4%) |
| CCCCCACAACCGCGCTTGACTAGCTTGCTGTTTT | 4.4% 4.2% |

## Slide 6
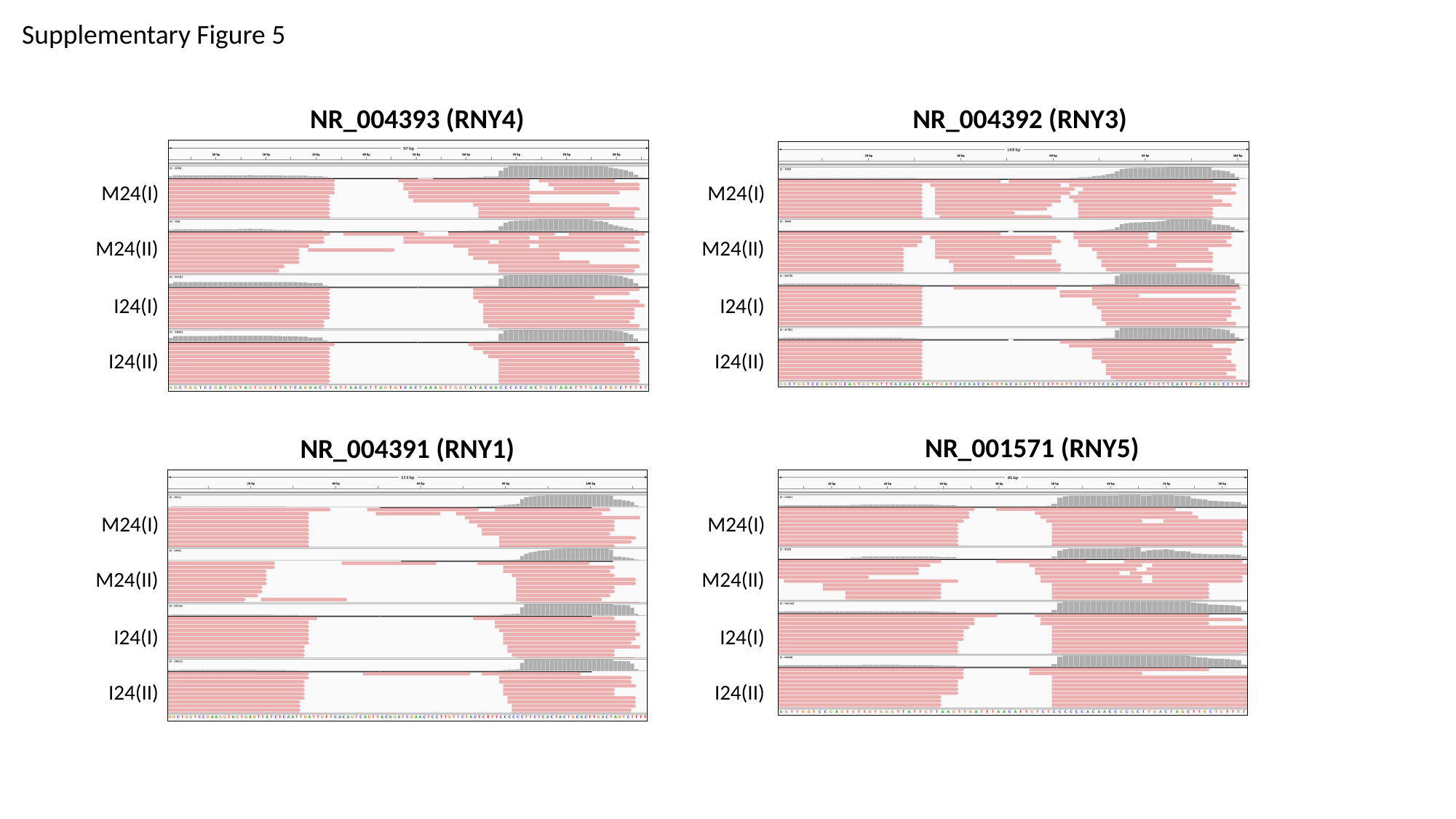

Supplementary Figure 5
NR_004393 (RNY4)
NR_004392 (RNY3)
M24(I)
M24(I)
M24(II)
M24(II)
I24(I)
I24(I)
I24(II)
I24(II)
NR_001571 (RNY5)
NR_004391 (RNY1)
M24(I)
M24(I)
M24(II)
M24(II)
I24(I)
I24(I)
I24(II)
I24(II)

## Slide 7
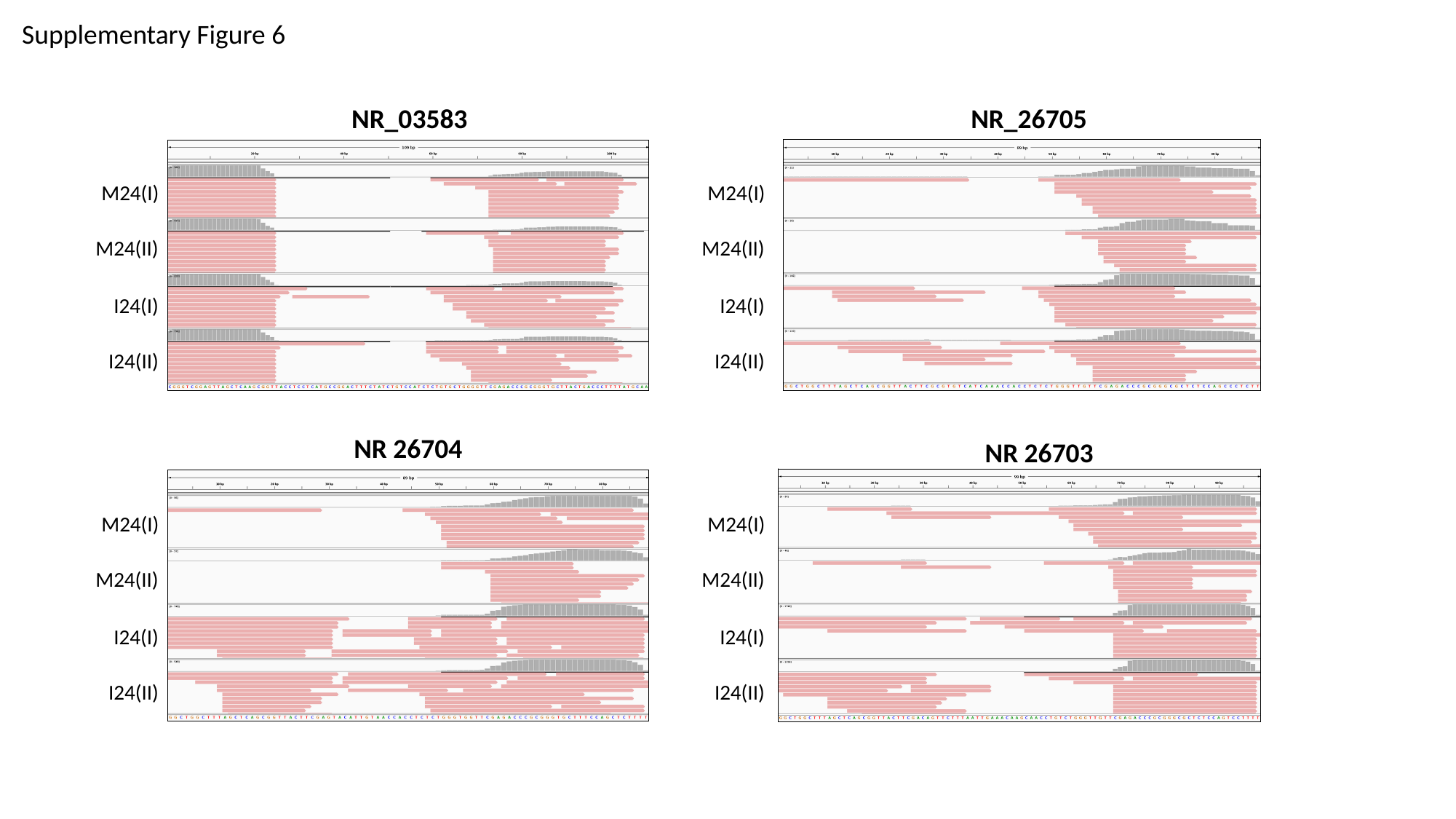

Supplementary Figure 6
NR_26705
NR_03583
M24(I)
M24(I)
M24(II)
M24(II)
I24(I)
I24(I)
I24(II)
I24(II)
NR 26704
NR 26703
M24(I)
M24(I)
M24(II)
M24(II)
I24(I)
I24(I)
I24(II)
I24(II)

## Slide 8
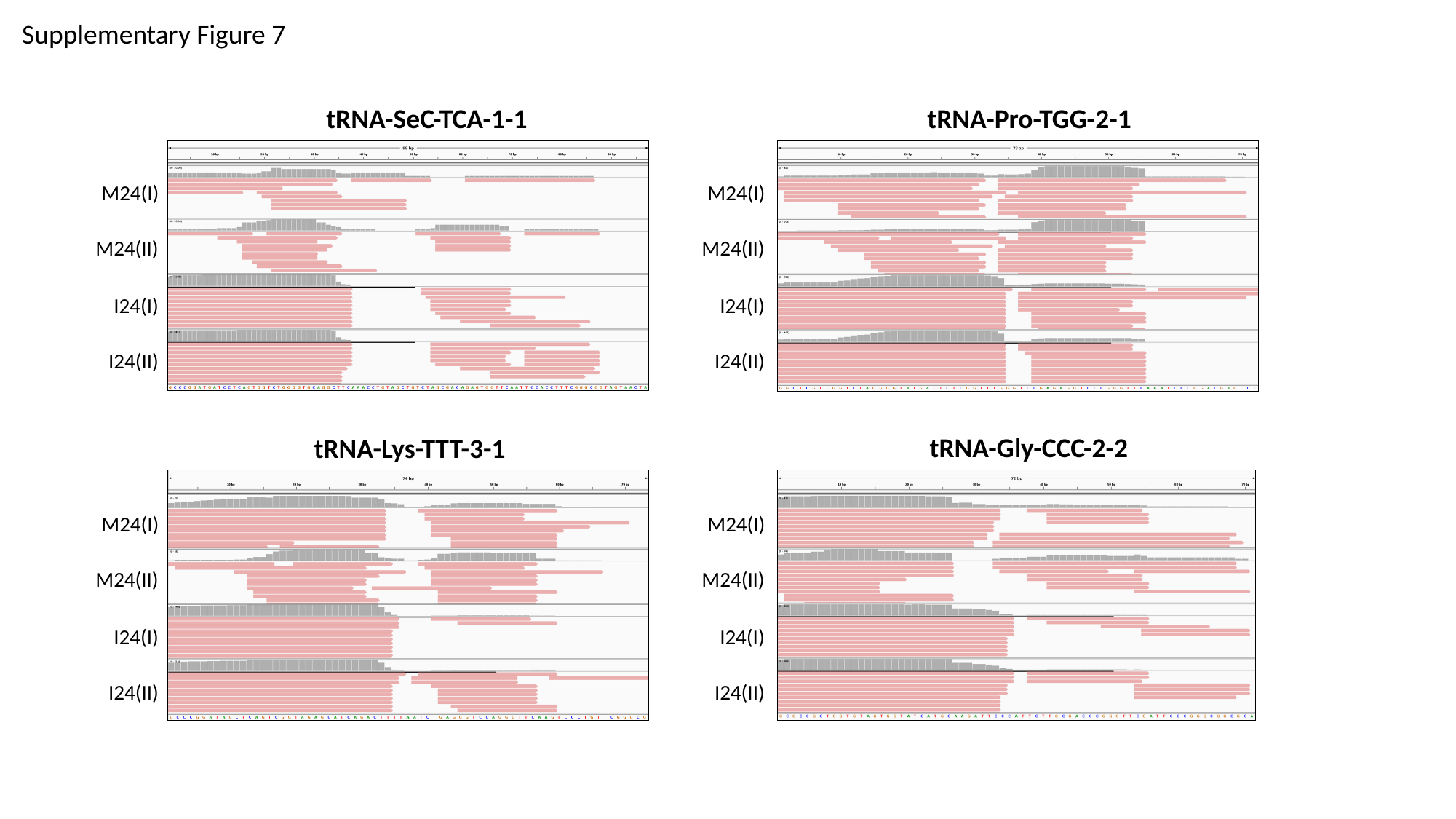

Supplementary Figure 7
tRNA-SeC-TCA-1-1
tRNA-Pro-TGG-2-1
M24(I)
M24(I)
M24(II)
M24(II)
I24(I)
I24(I)
I24(II)
I24(II)
tRNA-Gly-CCC-2-2
tRNA-Lys-TTT-3-1
M24(I)
M24(I)
M24(II)
M24(II)
I24(I)
I24(I)
I24(II)
I24(II)

## Slide 9
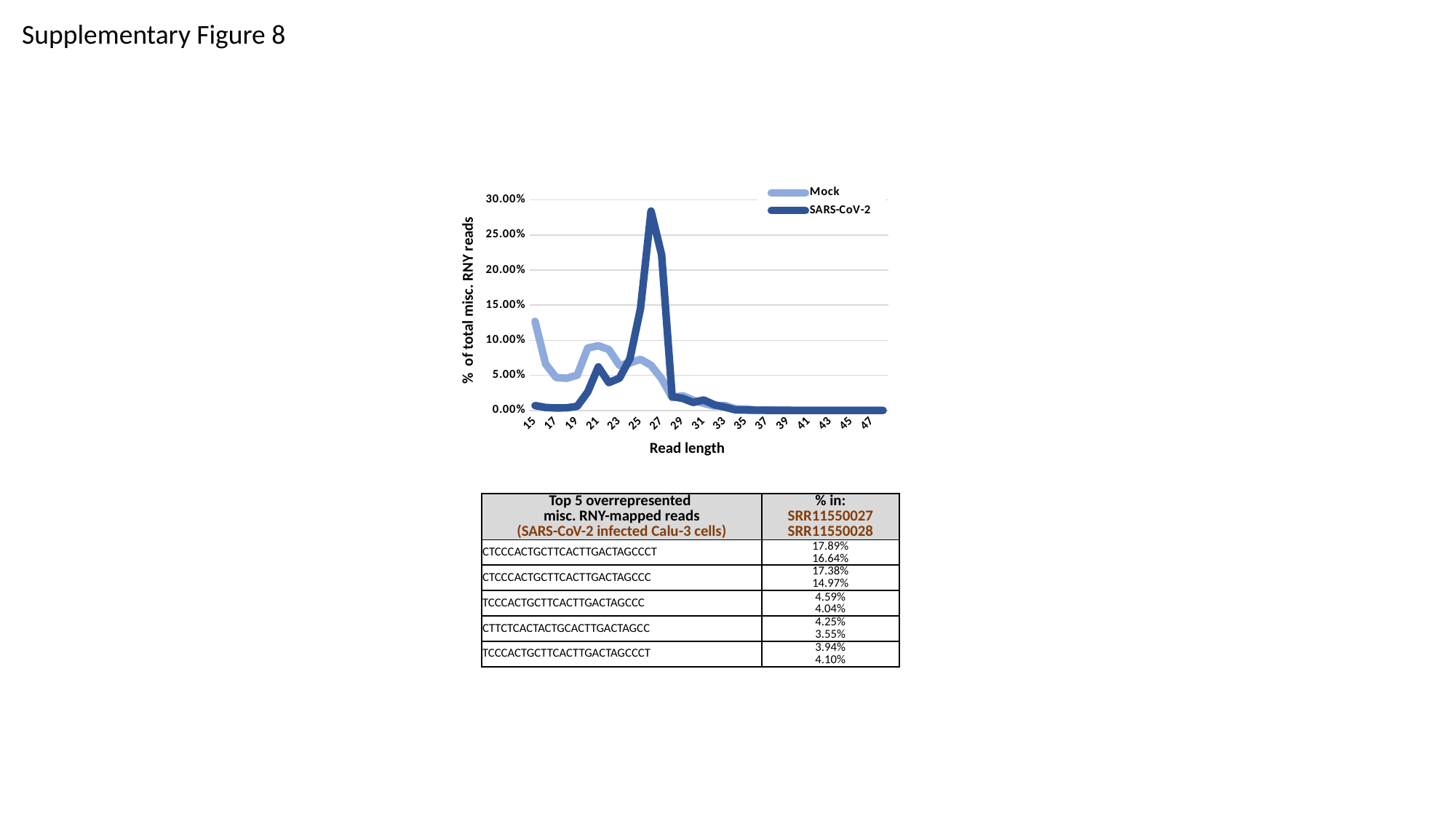

Supplementary Figure 8
### Chart
| Category | Mock | SARS-CoV-2 |
|---|---|---|
| 15 | 0.12706686724709831 | 0.006807547728858004 |
| 16 | 0.06609008559195369 | 0.004109743375064961 |
| 17 | 0.04686638931755448 | 0.003417082361792172 |
| 18 | 0.04578091227411436 | 0.003652967873006914 |
| 19 | 0.050293466219218014 | 0.005812706809173484 |
| 20 | 0.08887316740400746 | 0.02627913061586229 |
| 21 | 0.09219018941579465 | 0.061975443710350214 |
| 22 | 0.08679062138123741 | 0.03955857968644119 |
| 23 | 0.06429420828049755 | 0.0458995641595474 |
| 24 | 0.06757269085014975 | 0.07374796487209069 |
| 25 | 0.07286776743135934 | 0.14536794437791084 |
| 26 | 0.06424494657887092 | 0.284299956384199 |
| 27 | 0.045235998954478544 | 0.22194159081074857 |
| 28 | 0.01829278161306481 | 0.019642643136869392 |
| 29 | 0.02089343530674159 | 0.017093790833723147 |
| 30 | 0.014568111433812907 | 0.011334855186290141 |
| 31 | 0.00992881228924691 | 0.014798058858688118 |
| 32 | 0.006369426751592357 | 0.007969406083679378 |
| 33 | 0.007014077315178592 | 0.004605426114915968 |
| 34 | 0.0021433392110197314 | 0.0007801618485864836 |
| 35 | 0.0020606968902210565 | 0.0004768471926832595 |
| 36 | 0.00018733608092918696 | 0.00023137671570671404 |
| 37 | 0.0003746721618583739 | 0.00016304581191662658 |
| 38 | 0.0 | 2.2776967930029154e-05 |
| 39 | 0.0 | 1.1388483965014577e-05 |
| 40 | 0.0 | 0.0 |
| 41 | 0.0 | 0.0 |
| 42 | 0.0 | 0.0 |
| 43 | 0.0 | 0.0 |
| 44 | 0.0 | 0.0 |
| 45 | 0.0 | 0.0 |
| 46 | 0.0 | 0.0 |
| 47 | 0.0 | 0.0 |
| 48 | 0.0 | 0.0 |% of total misc. RNY reads
Read length
| Top 5 overrepresented misc. RNY-mapped reads (SARS-CoV-2 infected Calu-3 cells) | % in: SRR11550027 SRR11550028 |
| --- | --- |
| CTCCCACTGCTTCACTTGACTAGCCCT | 17.89% 16.64% |
| CTCCCACTGCTTCACTTGACTAGCCC | 17.38% 14.97% |
| TCCCACTGCTTCACTTGACTAGCCC | 4.59% 4.04% |
| CTTCTCACTACTGCACTTGACTAGCC | 4.25% 3.55% |
| TCCCACTGCTTCACTTGACTAGCCCT | 3.94% 4.10% |

## Slide 10
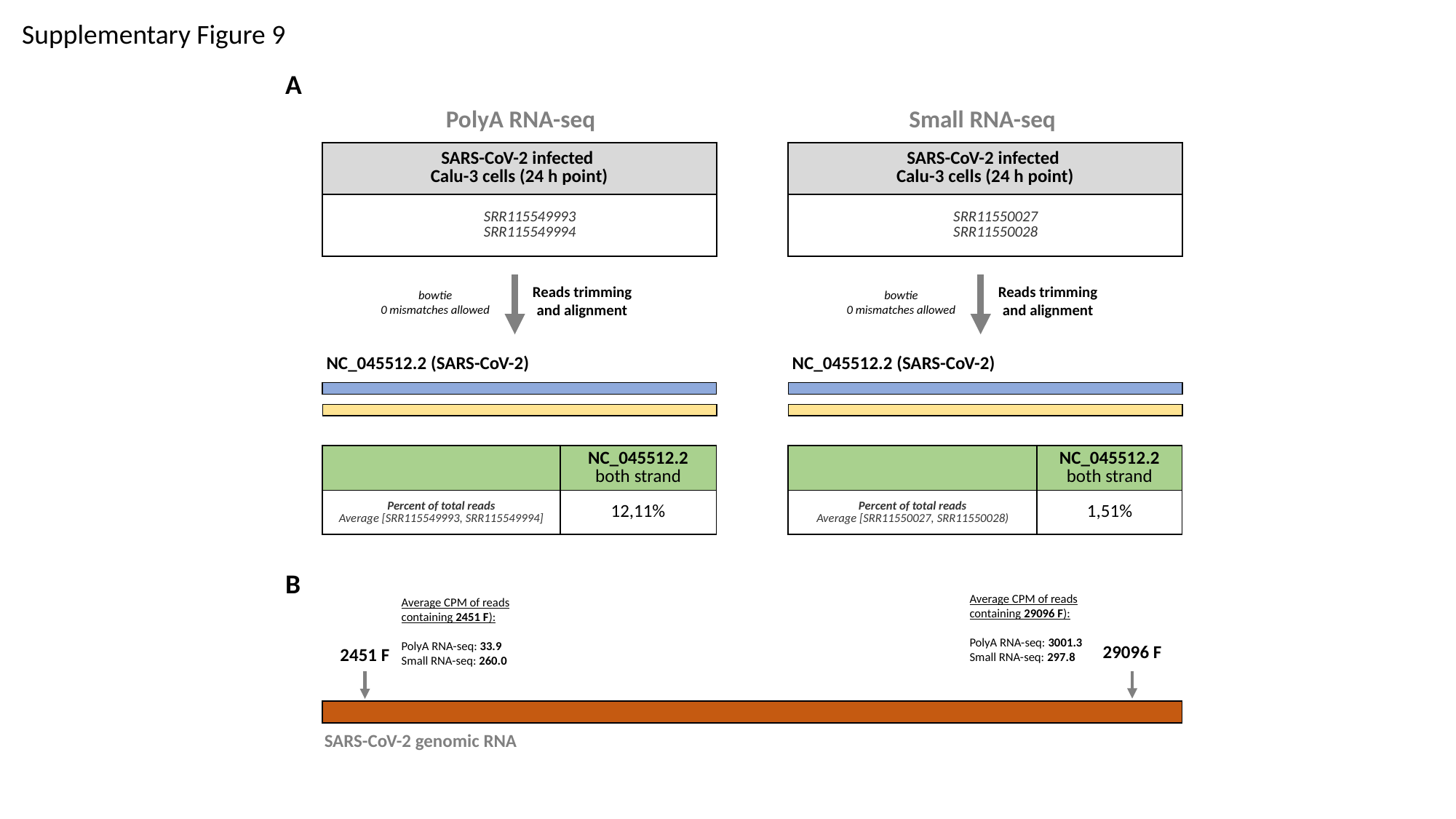

Supplementary Figure 9
A
PolyA RNA-seq
Small RNA-seq
| SARS-CoV-2 infected Calu-3 cells (24 h point) |
| --- |
| SRR115549993 SRR115549994 |
| SARS-CoV-2 infected Calu-3 cells (24 h point) |
| --- |
| SRR11550027 SRR11550028 |
Reads trimming
and alignment
Reads trimming
and alignment
bowtie
0 mismatches allowed
bowtie
0 mismatches allowed
NC_045512.2 (SARS-CoV-2)
NC_045512.2 (SARS-CoV-2)
| | NC\_045512.2 both strand |
| --- | --- |
| Percent of total reads Average [SRR115549993, SRR115549994] | 12,11% |
| | NC\_045512.2 both strand |
| --- | --- |
| Percent of total reads Average [SRR11550027, SRR11550028) | 1,51% |
B
Average CPM of reads containing 29096 F):
PolyA RNA-seq: 3001.3
Small RNA-seq: 297.8
Average CPM of reads containing 2451 F):
PolyA RNA-seq: 33.9
Small RNA-seq: 260.0
29096 F
2451 F
SARS-CoV-2 genomic RNA
